# Wax Protrusions on Anti-Adhesive Plant Surfaces and Their Interactions with Insect Adhesive Pads: A Mechanical Interpretation

**DOI:** 10.3390/biomimetics9070442

**Published:** 2024-07-19

**Authors:** Feodor M. Borodich, Zaida Gao, Elena V. Gorb, Stanislav N. Gorb, Xiaoqing Jin

**Affiliations:** 1 College of Aerospace Engineering, Chongqing University, Chongqing 400030, China; gaozd@cqu.edu.cn; 2 Department of Functional Morphology and Biomechanics, Zoological Institute, University of Kiel, Am Botanischen Garten 1-9, 24098 Kiel, Germany; egorb@zoologie.uni-kiel.de (E.V.G.); sgorb@zoologie.uni-kiel.de (S.N.G.)

**Keywords:** insect–plant interactions, surface, adhesion, bio-inspired technology

## Abstract

Insect attachment devices enhance adhesion to complex-geometry substrates by increasing the real contact area. In nature, insects mainly interact with plant surfaces that are often covered by 3D wax structures. Here, we describe, discuss, and give a mechanical interpretation of plant waxes and the possible fracture mechanisms of these wax structures during their interactions with the adhesive pads of insects. It is argued that these plant surface microstructures significantly influence insect adhesion through reducing the contact area and contaminating the insect pads.

## 1. Introduction

Exploring biomimetics in relation to insect attachment devices may help to prepare artificial adhesives with numerous attachment–detachment cycles. Indeed, in order to adapt to complex and changing environments, insects, geckos, tree frogs, and other animals have evolved complex micro- and nanostructures on their legs to control the adhesion function on various natural substrates. On the other hand, plants have developed some mechanisms to prevent insects from adhering to their surfaces. Many leaves and fruits are covered by crystalline wax structures that decrease insects’ abilities to adhere to these surfaces. Here, we discuss the mechanical properties of plant waxes and present a mechanical interpretation of the mechanisms of fracturing in these 3D wax projections. These models explain the microscopic mechanisms of insect attachment to plant surfaces from a multidisciplinary perspective, providing a theoretical basis for understanding the basic principles of biological attachment and transferring them to biomimetic applications. Hence, they may be used to explain the behavior of biological and artificial anti-adhesive surfaces with micro- and nanostructures. 

The animals mentioned above can modulate attachment strength via shear-sensitive adhesive pads and manage detachment by altering the angle of attachment of the limb to the substrate [1,2,3,4]. This rapid ability of organisms to establish and release attachment has significantly inspired research during last decades. Numerous researchers have elucidated the mechanisms behind biological climbing. When organisms respond to complex environments with different modes of locomotion, a combination of strong adhesion (attachment force perpendicular to a substrate) and strong friction (attachment force parallel to a substrate) is required [5,6]. Biological adhesion devices can be divided into two types: wet and dry. For example, spiders and geckos use dry adhesion, which is primarily achieved by intermolecular forces (Van der Waals) between deformable setae connected to the adhesive pad and the substrate [7,8,9]. Wet adhesion occurs when organisms secrete fluid on their feet and use capillary and viscous forces to adhere to the substrate [10,11,12]. Here, we concentrate on discussing the adhesion of insects, spiders, and geckos to surfaces contaminated by particulate materials of a certain shape, in particular by plant wax crystals.

In order to understand the mechanical principle of strong adhesion from some specialized plant surfaces, this review focuses mainly on exploring the interaction between the insect attachment organs and the plant surface. Plant surfaces exhibit a diverse array of textures in the form of micro- and nanostructures. These can be either smooth or structured, and the latter ones can be covered by different types of hairs (trichomes) or microscopic crystals of epicuticular waxes of very diverse shapes [13,14,15].

Plant crystalline waxes serve various functions, as discussed in reviews by Barthlott [16] and Bargel et al. [17]. Specifically, they protect plants by inhibiting insect attachment to their surfaces. This is important because the majority of insect species interact with plants, and therefore they typically need to adhere effectively to plant surfaces [18,19,20].

It is known that plant waxes are made up of a number of chemical substances [21,22,23]. Understanding the physical and chemical properties of plant waxes helps to improve our understanding of the mechanisms behind the anti-insect-attachment ability of plant surfaces. On one hand, understanding the rapid and reversible attachment mechanisms in biological systems creates new opportunities for the development of biomimetic applications, such as climbing robots, grippers, green adhesives, etc. On the other hand, understanding the effective anti-adhesive mechanisms of plant surfaces might help in the development of novel green anti-adhesive coatings or switchable controllable attachment devices employing switchable changes in the surface microstructure, as described in [24,25]. 

## 2. Plant Waxes, Their Properties, and Fracture Behavior

### 2.1. Structure of Plant Waxes

**Figure 1:**
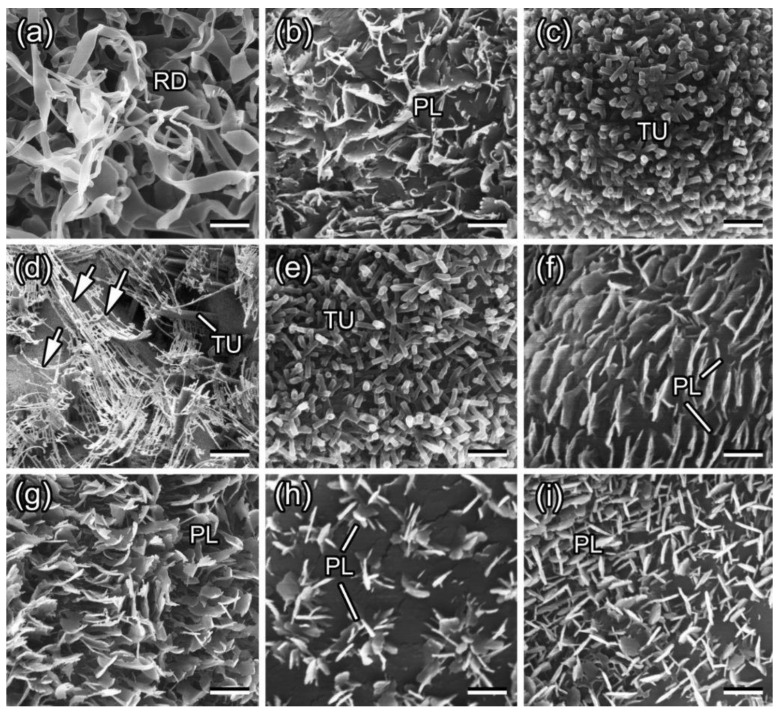
Scanning electron microscopy (SEM) micrographs of waxy plant surfaces in a young stem of *Acer negundo* (**a**) and in adaxial (upper) leaf sides of *Aloe vera* (**b**), *Aquilegia vulgaris* (**c**), *Brassica oleracea* (**d**), *Chelidonium majus* (**e**), *Chenopodium album* (**f**), *Iris germanica* (**g**), *Lactuca serriola* (**h**), and *Trifolium montanum* (**i**). PL, wax platelets; RD, wax rodlets; TU, wax tubules. Arrows in (**d**) denote filament-like branches on top of the tubules. Scale bars: 2 μm (**a**,**b**,**d**,**g**,**h**) and 1 μm (**c**,**e**,**f**,**i**). Reproduced with permission from [32].

Plants have developed cuticles to protect their internal tissues. These cuticles exhibit complex ultrastructures and chemical compositions in response to various environmental stresses and interactions with microorganisms, insects, and other abiotic and biotic factors. The cuticle may be impregnated with intracuticular waxes, or waxes may be transported across the cuticle and deposited on its surface as epicuticular waxes. Epicuticular waxes accumulate in forms ranging from amorphous films to microcrystalline structures [13]. Electron microscopy and X-ray diffraction analyses [13,21,26,27] have revealed the diverse structures of epicuticular waxes, such as massive crusts, filaments, rodlets, plates, etc. (Figure 1). The diversity of these shapes arises from molecular self-assembly on the cuticle surface [26,28,29,30,31].

**Figure 2:**
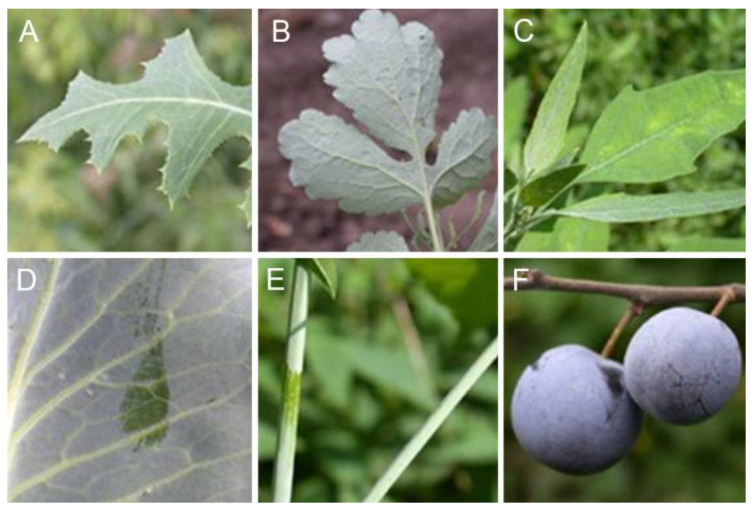
Surfaces of plants covered by epicuticular wax projections: (**A**) *Lactuca serriola*; (**B**) *Chelidonium majus*; (**C**) *Chenopodium album*; (**D**) *Brassica oleracea*; (**E**) *Acer negundo*; (**F**) *Prunus domestica*.

Epicuticular waxes include several major classes of alicyclic and long-chain aliphatic compounds, typically with homologous chain lengths in the range of C_16_ to C_35_ [21,22]. Waxes exhibit differences in their composition, abundance, relative distribution of classes, and homolog chain lengths, which vary among plant species, plant parts, developmental stages, and environmental factors. Microscopically small crystals frequently protrude from the wax film, which overlays the plant cuticle, giving rise to the pruinose or powdery appearance observed on the surfaces of numerous plant species. The length of these wax crystals ranges from a few hundred nanometers to several micrometers. These pruinose waxy surfaces are present on the stems, leaves, flowers, seeds, and fruits of numerous plant species (Figure 2).

**Figure 3:**
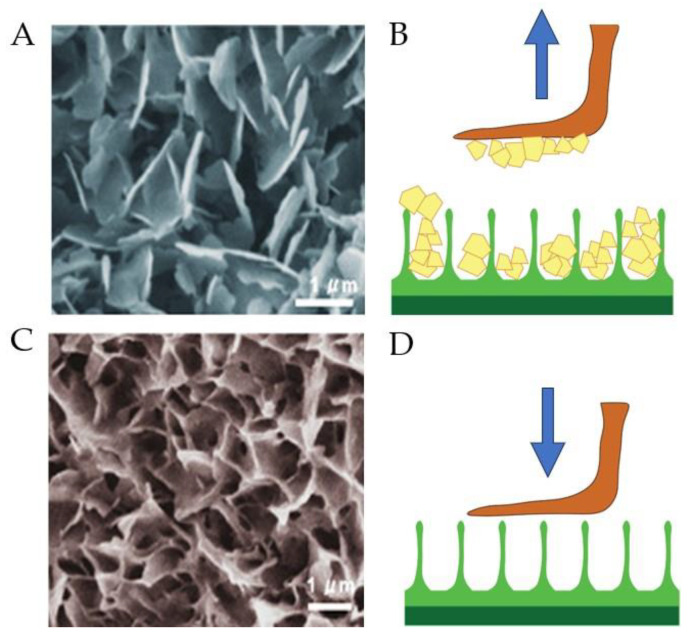
Layering of wax crystals of the pitcher plant Nepenthes alata reduces insect attachment capacity: (**A**) SEM image of epicuticular wax of the upper layer; (**B**) schematic image of contamination of an insect adhesive microstructure (red) by wax crystals of the upper wax layer (yellow); (**C**) SEM image of epicuticular wax of the lower layer; (**D**) schematic image of interaction between an insect adhesive microstructure and micro-roughness of a plant. Reproduced with permission from [33].

The structure of the epicuticular wax on Nepenthes alata comprises two distinct layers and warrants a detailed examination. The upper layer is composed of separate, easily distinguishable, irregular platelets that cover the surface [13] (Figure 3A). The crystals in the upper layer are brittle and can easily exfoliate or break into tiny pieces. Both whole crystals and small fragments can adhere to insect feet (Figure 3B), contaminating the attachment organs and impeding proper contact between adhesive pads and plant surfaces, which significantly reduces the attachment force. The lower layer is composed of interconnected membranous platelets resembling a foam (see Figure 3C). The crystals in the lower layer are not easily detached and can remain intact even after the removal of the upper layer. The crystal network can withstand lateral forces from climbing insects. However, its micro-roughness significantly decreases the contact area with the insects’ adhesive organs, as demonstrated in Figure 3D. This reduces the insect’s ability to attach.

### 2.2. Mechanical Properties of Plant Waxes

The following section provides a discussion of the mechanical properties of plant waxes. Unfortunately, the available experimental information about wax stress–strain curves is rather limited, even in the case of axially loaded samples. To the best of our knowledge, there is no available information about multiaxial stress states. It is understood that for moderate loads, specifically when the applied stresses are equal to a specific stress, the one-dimensional tension–compression proportional limit $\sigma_{pr}$ of crystalline materials obeys Hooke’s law, which establishes a linear relationship between stress σ and strain ε, or between tensile elongation or reduction δ and the force P applied to a tested specimen:

(1) $$\delta =\frac{PL}{AE}\;\mathrm{or}\;\sigma =E\varepsilon$$

where A represents the cross-sectional area of the specimen, E denotes a material property (Young’s modulus or elastic modulus), and L is the length of the sample. In a one-dimensional problem, the strain ε is calculated as δ/L, and σ is calculated as P/A.

**Figure 4:**
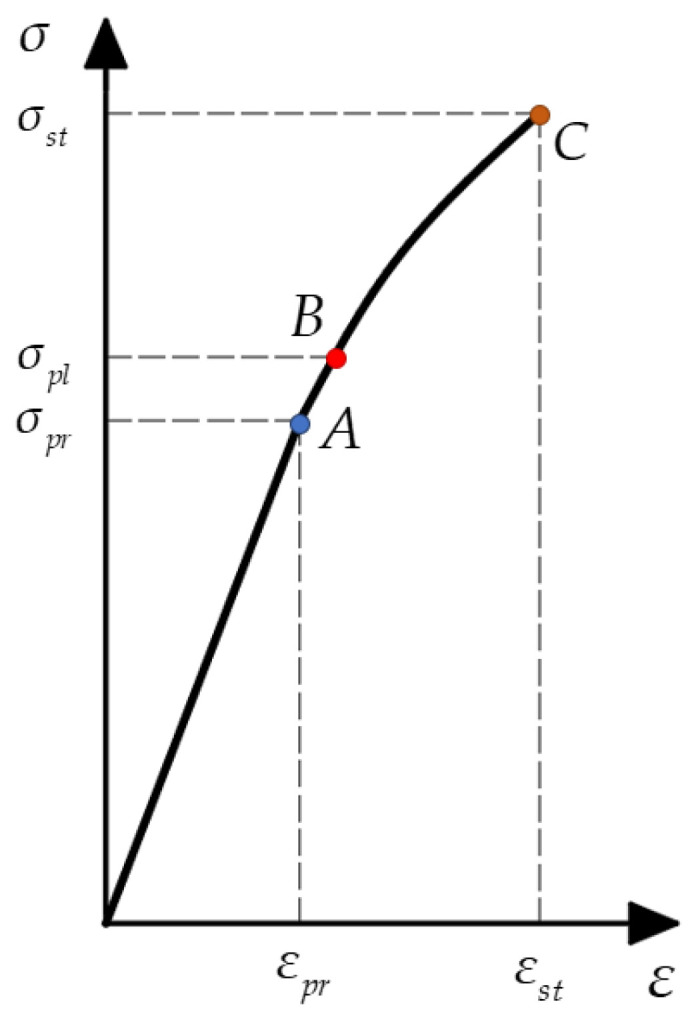
Schematic typical stress–strain diagram. Point A represents the proportional limit $\sigma_{pr}$, point B indicates the yield point $\sigma_{pl}$, and point C signifies the fracture stress $\sigma_{st}$ (the material’s strength).

When stresses fall between the proportional limit $\sigma_{pr}$ and the yield stress $\sigma_{pl}\;(\sigma_{pr}\leq \sigma <\sigma_{pl})$, a small region of nonlinear elastic behavior may be observed. This means that after unloading, the sample retains its original shape, although the material’s behavior deviates from the linear relation. If the tensile (or compressive) stress exceeds the yield stress $\sigma_{pl}$, the sample undergoes plastic deformation upon unloading, resulting in a shape different from its pre-loading state. Typically, $\sigma_{pr}≅\sigma_{pl}$, suggesting they are equivalent. Brittle materials fail with minimal elongation or reduction (just a few percent) post yield stress (point B in Figure 4). Figure 4 depicts the standard stress–strain curve for a brittle crystalline material.

It has been observed that mechanical properties such as the Young’s modulus E, proportional limit $\sigma_{pr}$, and compressive strength $\sigma_{st}$ of waxes are generally temperature-dependent. The details of these are shown in Table 1.

For example, *carnauba* wax, which is extracted from the leaves of the *carnauba* wax palm (*Copernicia prunifera*), exhibits this temperature sensitivity. It has a Poisson’s ratio ν=0.49 and a density $\rho =951\;\mathrm{kg}/m^{3}$. Craig et al. [34] noted that the strain before fracture under compression for all tested waxes ranged from 2.7% to 4.3%. The samples exhibited brittle fracture behavior. This observation of the brittle fracture characteristics of crystalline waxes aligns with the findings from experiments on plant waxes reported in [33]. Shellhammer et al. [35] found that natural *candelilla* and *carnauba* waxes behaved similarly to hard and elastic materials at 2% compressive strain, with *candelilla* wax exhibiting greater viscosity than *carnauba* wax. However, both waxes demonstrated behavior more akin to that of elastic materials compared to that of beeswax, which exhibited significantly more viscosity and less elastic behavior.

The widespread claim that *carnauba* wax is the hardest known wax is supported by evidence that it has the highest mechanical properties at the relevant temperature of all of the waxes tested by Craig et al. [34]. However, our previous experiments [33] indicated that other plant waxes may exhibit higher hardness and elastic modulus values compared to *carnauba* wax. Specifically, depth-sensing nanoindentation on the wax of the carnivorous plant Nepenthes alata revealed an elastic modulus of approximately E=2.5 GPa and a hardness of H=0.1 GPa at T=22 °C. 

The elasticity of real materials significantly affects the elastic–plastic indentation process. Initially, when the yield point is exceeded, the plastic zone is small and completely surrounded by material that remains elastic. This results in plastic strains that are comparable to the surrounding elastic strains. In such cases, the material displaced by the indenter is absorbed by the elastic expansion of the surrounding solid. With increasing indentation depth, the plastic zone eventually extends to the free surface, allowing displaced material to flow plastically to the sides of the indenter. Johnson [36] suggested the following relation between the mean pressure $p_{m}$ acting normal to the original surface as $p_{m}=c\sigma_{pl}$, where the constant c, which depends on the geometry of the indenter and the interfacial friction, typically has a value of about 3. Initially, when yielding first occurs, the constant c is approximately one. In the mechanics of elastic–plastic contact, it is generally accepted that hardness is the average stress under the indenter at which the entire material yields. Using Johnson’s calculation method, one can approximate $H\approx c\sigma_{pl},\;2.8\leq c\leq 3$. Hence, we can estimate for the wax of the carnivorous plant Nepenthes alata, having *H* = 0.1 GPa at *T* = 22 °C, that $\sigma_{pl}$ is approximately 35 MPa. 

**Figure 5:**
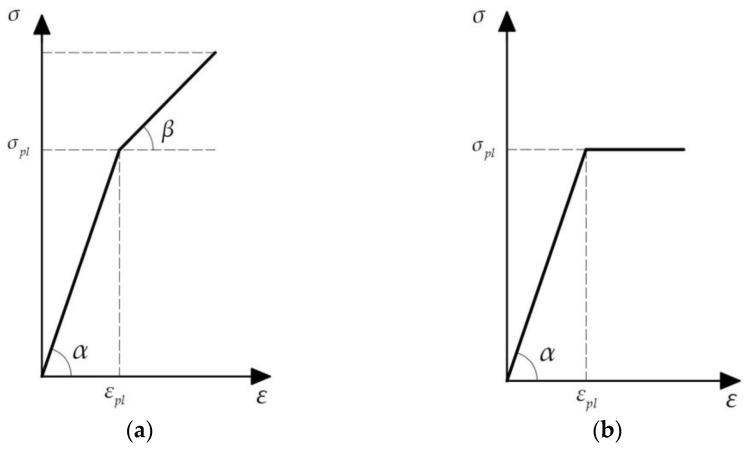
(**a**) Bilinear stress–strain diagram and (**b**) linear elastic–ideal plastic diagram. It is noted that tanα=E and $\tan\beta =E_{1}$, where E and $E_{1}$ are Young’s modulus and the plastic hardening modulus, respectively.

We can assume that the waxes have a bilinear diagram (Figure 5), whose reference points A, B, and C vary with temperature. Figure 5 depicts an idealized stress–strain diagram in two cases: (a) a bilinear diagram with strain hardening, and (b) linear elastic–ideal plastic diagram. When stresses have absolute values less than the yield stress $\sigma_{pl}$, the material obeys Hooke’s law (1) with E=tanα. If the absolute value of stress exceeds the yield stress, strain hardening is approximated by a line with another slope: $E_{1}=\tan\beta$. If β=0, then $\sigma =\sigma_{pl}$. This indicates that the wax yields without any further increase in the external load. With Hooke’s law, calculations can proceed as follows:

(2) $$\varepsilon_{pl}=\frac{\sigma_{pl}}{E}=\frac{35\cdot 10^{6}}{25\cdot 10^{9}}=0.014$$

Based on the experiments conducted by Craig et al. [34], it can be assumed that the strain at fracture is 2.7%, i.e., $\varepsilon_{st}=0.027$. To plot the bilinear diagram, we need either the slope of the linear hardening part (the tangent modulus $E_{1}$) or the stress at fracture $\sigma_{st}$. From this bilinear diagram, we derive the relation $\varepsilon_{st}-\varepsilon_{pl}=\left(\sigma_{st}-\sigma_{pl}\right)/E_{1}$. 

We may assume that the ratio $\sigma_{st}/\sigma_{pl}$ of the Nepenthes alata wax is the same as that of carnauba wax, i.e., 1.575. Therefore, we obtain $\varepsilon_{st}-\varepsilon_{pl}=0.013=0.575\sigma_{pl}/E_{1}$. Thus, $E_{1}$ can be calculated as $E_{1}=20.125\cdot 10^{6}/0.013=1.548\cdot 10^{9}\;\mathrm{Pa}$.

### 2.3. Deformation and Fracture of Plant Wax Structures

#### 2.3.1. Euler Buckling of Wax Projections

**Figure 6:**
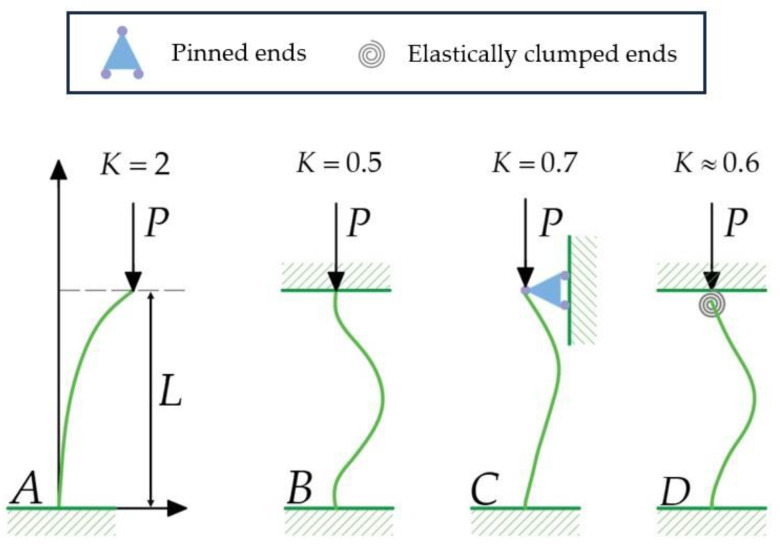
Some boundary conditions for compressed columns: (**A**) a column with built-in and free ends; (**B**) a column with both ends built-in; (**C**) a column with built-in and pinned ends; (**D**) a column with built-in and elastically clumped ends. K is the effective length factor for different boundary conditions at the end of the column.

In the mechanics of materials, long and slender structures, as plant wax projections, subjected to axial compression are referred to as columns. First, let us analyze the stability of tubular columns, which resemble tubular wax crystals, under compressive loading. When the applied compressive load P increases, a slender elastic column will buckle at the critical load $P_{cr}$, determinable using the Euler formula:

(3) $$P_{cr}=\frac{\pi^{2}EI}{{\left(KL\right)}^{2}}=\frac{\pi^{2}EI}{{\left(L_{ef}\right)}^{2}}$$

where K represents the effective length factor, which varies depending on the boundary conditions at the end of the column, and where effective length is defined as $L_{ef}=KL$. When a column has pinned ends, K is 1. When the base is built-in (fixed) and the top end is free (Figure 6A), K is 2. When the base is fixed and the top is pinned (Figure 6C), K is 0.7. When both ends are built-in (Figure 6B), K is 0.5. It is plausible to assume that one end of the wax projection is attached to the plant surface, indicating a built-in state, while the other end is involved in adhesive interactions with the insect’s attachment organ (adhesive pad). Insects usually rely on capillary adhesion resulting from the secretion of fluid (pad secretion) into the contact zone between the pad and the substrate [10]. These conditions suggest that the column may be modeled as a rod with an elastically restrained (clumped) end, which may be represented as an elastic torsional spring at this end (Figure 6D). The torsion spring model represents the adhesive interaction between an insect’s seta and a wax column. Consequently, $L_{ef}=0.6L$ can be considered a reasonable approximation for the effective length of the column.

#### 2.3.2. Elastoplastic Buckling of Wax Columns

The Euler formula in Formula (4) assumes a linearly elastic stress–strain relationship and becomes invalid when compressive stresses in the column exceed the yielding stress. Consequently, inelastic buckling must be considered. Engesser and Jasinski developed this theory, as cited in [37]. According to this theory, the critical load $P_{cr}$ at which a slender elastic–plastic column buckles can be calculated as follows:

(4) $$P_{cr}=\frac{\pi^{2}E_{r}I}{{\left(KL\right)}^{2}}$$

where $E_{r}$ represents the column’s reduced modulus. For a rectangular cross-section, we can calculate this modulus using Formula (5). This serves as an approximation for columns with circular cross-sections.

(5) $$E_{r}=\frac{4EE_{1}}{{\left(\sqrt{E}+\sqrt{E_{1}}\right)}^{2}}$$

Given the moduli of elasticity, E=2.5 GPa and $E_{1}=1.548\;\mathrm{GPa}$, for the plant wax, the reduced modulus of the column can be calculated as follows:

(6) $$E_{r}=\frac{4\cdot 2.5\cdot 1.548}{{\left(\sqrt{2.5}+\sqrt{1.548}\right)}^{2}}=1.94\;\mathrm{GPa}$$

These formulas are applicable to slender columns. To characterize the behavior of a column, it is essential to introduce the slenderness ratio $\lambda =L_{ef}/r$, where $r=\sqrt{I/A}$, the radius of gyration of the column’s cross-section in the bending plane. Here, A denotes the cross-section’s area, and I represents the moment of inertia for the cross-sectional area. For a circular cross-section with diameter D, $A=\pi D^{2}/4$ and $I=\pi D^{4}/64$, the radius of gyration is calculated below:

(7) $$r=\sqrt{\frac{I}{A}}=\sqrt{\frac{4D^{4}}{64D^{2}}}=\frac{D}{4}$$

It is important to understand the difference between the aspect ratio and the slenderness of a column. The aspect ratio is a purely geometric characteristic, defined as the ratio between the largest and smallest dimensions of the column, whereas the slenderness depends on both the geometry, represented by the length L and the radius of gyration r of the column’s cross-section, and the loading conditions, indicated by the effective length factor K. For instance, although all cases A–D in Figure 6 share the same aspect ratio L/D, their slenderness values differ significantly. The Euler formula in Formula (3) is applicable solely to purely linearly elastic materials; therefore, the transition from elastic to the elastic–plastic case, i.e., from Formula (4) to Formula (3), should be at the critical force such that it causes the critical stress to be equal to the yield stress, as follows:

(8) $$\sigma_{cr}=\frac{P_{cr}}{A}=\frac{\pi^{2}EI}{AL_{ef}^{2}}=\sigma_{pl}$$

Consequently, the critical slenderness ratio can be calculated as follows:

(9) $$\lambda_{c}={\left(\frac{L_{ef}}{r}\right)}_{c}=\sqrt{\frac{\pi^{2}E}{\sigma_{pl}}}$$

**Figure 7:**
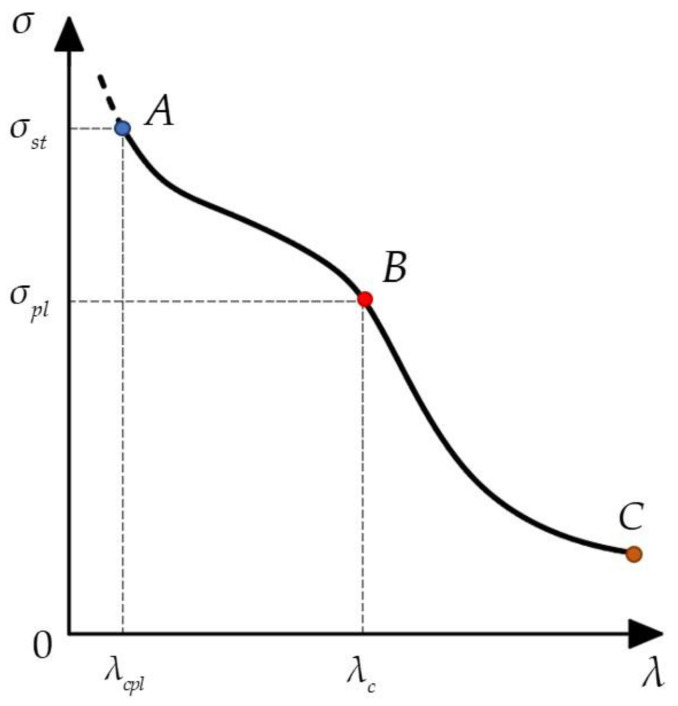
Relationship between plant wax compressive stress and slenderness ratio. In region BC, the critical stress at the yield limit $\sigma_{pl}$ is given by $\sigma_{cr}=\frac{\pi^{2}E}{\lambda^{2}}$. In region AB, the critical stress at the strength limit $\sigma_{st}$ is given by $\sigma_{cr}=\frac{\pi^{2}E_{r}}{\lambda^{2}}$. When $\lambda \leq \lambda_{cpl}$, the material will not buckle, as it has already exceeded the strength limit, and will instead be crushed.

Hence, the relationship between the average compressive stress σ and slenderness ratio λ for plant waxes may be represented by the graph shown in Figure 7.

Substituting the values for plant wax, we obtain the following critical slenderness ratio:

(10) $$\lambda_{c}=\sqrt{\frac{9.87\cdot 2.5\cdot 10^{9}}{35\cdot 10^{6}}}=26.5$$

Hence, the slenderness ratio for columns with K=0.6 and gyration radius r=D/4 is as follows:

(11) $$\lambda =\frac{0.6L}{r}=\frac{2.4L}{D}$$

**Figure 8:**
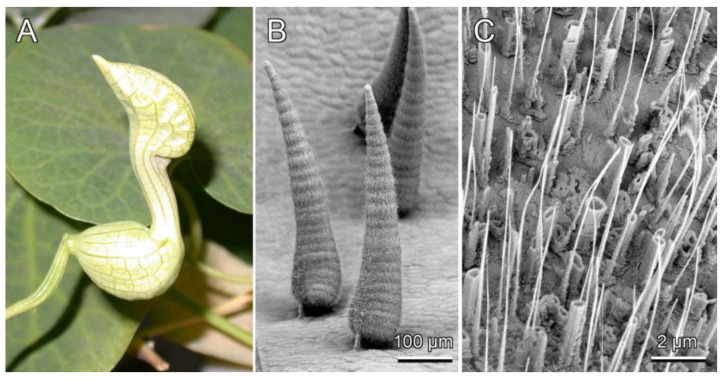
(**A**) White-veined Dutchman’s pipe (*Aristolochia fimbriata*) flower; (**B**) scanning electron microscopy micrographs of trichomes on the inner surface of the flower trap; (**C**) wax crystals covering the trichome surface that may buckle.

Gorb et al. [38] report that certain plants, such as *Aquilegia vulgaris* (European columbine), *Berberis vulgaris* (common barberry), *Chelidonium majus* (white goosefoot), and *Prunus domestica* (European plum), exhibit tubular epicuticular wax crystals. *Aristolochia fimbriata* (white-veined Dutchman’s pipe) crystals, as well as those of many other *Aristolochia* species, also exhibit this tubular shape, but at a very high slenderness ratio, as shown in Figure 8. Table 2 presents the average lengths and diameters of these crystal columns, along with the calculated slenderness ratios ($\lambda =L_{ef}/r$) for all five plants mentioned above.

Clearly, among the tubular-shaped plant wax crystals studied [39,40], only *Aristolochia fimbriata* can elastically buckle, as the slenderness ratios for the other plants fall below the critical value of 26.5. 

Therefore, the crystals from these four plants do not meet the length criteria for the Euler formula approximation. Formula (3) applies solely under stresses below the material’s ultimate compressive stress $\sigma_{st}$. Consequently, the critical slenderness ratio $\lambda_{cpl}$ for discontinuing the use of Formula (3) due to elastic–plastic buckling is identified as follows:

(12) $$\lambda_{cpl}={\left(\frac{L_{ef}}{r}\right)}_{cpl}=\sqrt{\frac{\pi^{2}E_{r}}{\sigma_{st}}}$$

The plant critical slenderness ratio $\lambda_{cpl}$ can be obtained by substituting the specified plant wax values into Formula (12), as follows:

(13) $$\lambda_{cpl}=\sqrt{\frac{9.87\cdot 1.94\cdot 10^{9}}{55\cdot 10^{6}}}=18.66$$

Plotting a diagram of the average stress against the slenderness ratio (Figure 7) reveals that, aside from *Aristolochia fimbriata*, which buckles in the elastic regime, crystals from all other studied plants do not buckle in either the elastic or elastic–plastic regimes given the calculated slenderness ratios. Indeed, these short columns are not susceptible to failure through a pure buckling mechanism. Failure occurs only when compressive stress reaches the wax’s strength limit. Therefore, it is necessary to account for the bending of wax crystals under simultaneous axial and orthogonal loading.

There are two restrictions in the application of the above Euler formula: One is that the default compression column is perfectly straight before the load is applied, but in reality, the presence of tubular columnar plant wax crystals does not guarantee perfect straightness. Secondly, Euler’s formula assumes that the applied load passes exactly through the centroid of the compressed column cross-section, but in reality, the applied load will always deviate slightly from the centroid of the cross-section. While the Euler formula provides a useful framework for understanding the behavior of compressed columns, its assumptions may not fully capture the complexities of real-world scenarios, particularly when dealing with tubular columnar plant wax crystals. As highlighted, the assumption of perfect straightness before the application of load overlooks the inherent imperfections present in natural structures. These discrepancies underscore the need for a more detailed consideration of the loading schemes of such structures. Transitioning from the limitations of Euler’s formula, our study delves into the realm of the elastoplastic bending of beams.

#### 2.3.3. Bending of Wax Beams: Elastoplastic Bending

We aim to provide a more accurate understanding of the mechanical response of columnar plant wax crystals under various loading conditions and aspect ratios.

**Figure 9:**
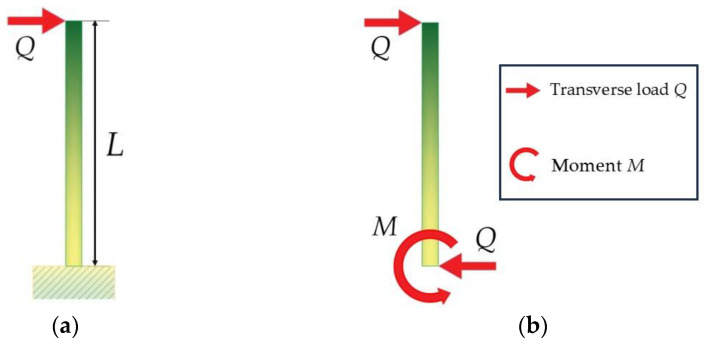
(**a**) A built-in wax rod (length *L*) under the action of a transverse load *Q*; (**b**) free-body diagram for this wax rod (length *L*) under the action of a transverse load *Q* and moment M.

Let us consider the case in which the wax material exhibits linear elastic and ideal plastic behavior (Figure 9b). Hence, the beam material is assumed to obey Hooke’s law up to the yield stress $\sigma_{pl}$, beyond which it deforms plastically without any hardening. 

So far, we have considered only loads acting along the axis of the wax structure (the rod of length *L*). If the load is inclined, then it can be decomposed into the vertical load P and the component Q, perpendicular to the axis of the rod. Let us consider now the results of the actions of the force Q. If the rod is built-in as part of the cuticle, then it can be modeled as a cantilever beam (see its free body diagram in Figure 9). 

**Figure 10:**
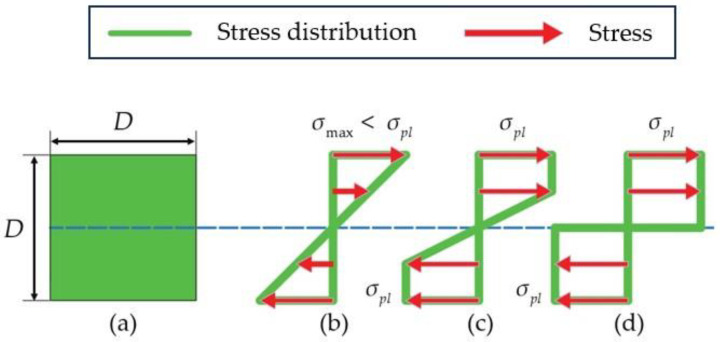
Stress distribution. (**a**) Cross-section of the rod (the side length of a cross-square section is *D*); (**b**) the bending stress distribution in the cross-section at the built-in end when $\sigma_{\max}<\sigma_{pl}$; (**c**) the stress distribution when stresses in some fibers reach the plastic limit; (**d**) the plastic hinge stresses.

To illustrate the main idea, we will consider a simple case with a constant square cross-section of the rod. Let the size of the square have an edge length *D* (see Figure 10a). Initially, the wax exhibits linear elastic behavior and the stress distribution is linear, and the maximum stress at the built-in end is $\sigma_{\max}=M\cdot D/2I,\;I=D^{4}/6,\;M=Q\cdot L$ (see Figure 10b). However, the maximum stress cannot be greater than $\sigma_{pl}$. Hence, if Q increases, then the stress distribution becomes as it is shown in Figure 10c. As soon as Q reaches the value $Q=\sigma_{pl}D^{3}/4L$, a plastic hinge (see Figure 10d) appears in the built-in cross-section, and the rod breaks. Similar calculations can be carried out for tubular cross-sections of rods.

Let us consider an example [39]. It is assumed that the example insect (a syrphid fly) has a mass of $160\cdot 10^{-6}\mathrm{kg}$. During locomotion, the syrphid fly’s weight is distributed across half of its legs, resulting in an approximate load of 523.2 nN per foot. Each fly foot consists of two pulvilli with hairy adhesive pads. Such structures help the insect to increase the actual area of its attachment to the counterpart surface. The force exerted by each bristle on a wax crystal, calculated from the density of the bristles on the adhesive pads, is G=157.5 nN. When the stress in the beam’s outermost fiber meets the yield stress σ, this is defined as the plastic yield moment, $M_{pl}$. For a beam of a square cross-section with dimension D, $M_{pl}$ is calculated as follows:

(14) $$M_{pl}=\frac{\sigma_{pl}D^{3}}{6}$$

For the five plant wax crystals mentioned above, each has a different size D, and the corresponding yield moments $M_{pl}$ are given in Table 3 below.

If the transverse component of the applied force is Q, i.e., if Q is the force applied transversely to the crystal axis, then the bending moment is M(L)=QL. If the plant surface has an angle θ with the horizontal plane, then Q=Gsinθ. If θ=90°, then G=157.5 nN. By calculation, the bending moment value $M_{L}$ on the wax crystal can be obtained as shown in Table 4.

In the most straightforward instance of inelastic bending, known as ideal plastic bending, the plastic hinge assumes that materials yield plastically under a constant stress (Figure 10d). In this model, plastic yielding starts at the fibers furthest from the neutral axis (Figure 10b). As the bending moment increases, the plastic region expands further inwards (Figure 10c). The beam reaches its maximum moment-resisting capacity when the entire cross-section enters the plastic region (Figure 10d). This specific moment is called the plastic moment $M_{p}$.

The ratio of a beam’s plastic moment to its yield moment, which is determined solely by the shape of the cross-section, is known as the shape factor *f*. For a circular cross-section, f is 1.7; for a square cross-section, f is 1.5. After calculation, the plastic moment $M_{p}$ for a square cross-section beam is given as the value shown in Table 3. Upon comparison, it can be seen that for all of the plant crystals considered, except *Prunus domestica*, the ultimate bending moment values $M_{p}$ that insects can produce are higher than the corresponding ultimate plastic moment values $M_{L}$, in which case the crystals are destroyed.

## 3. Conclusions

The relationship between the chemical composition and the morphology of wax crystals is rather well studied [41,42,43,44]. Shepherd et al. [45,46] detected unusual triacylglycerols in small amounts on leaves frequented by raspberry aphids. They reported that these compounds originated from the aphid cuticle on the leaf surface. Research by Matas et al. [47] has suggested that the diffusion or sorption of other molecules may disrupt the molecular arrangement of plant wax. Furthermore, hydrogen bonds may form between wax molecules and other molecules with different structures and functional groups. Van der Waals and hydrogen bond interactions can irreversibly disrupt the structure and morphology of some plant wax molecules. As is well known, insects can firmly adhere to natural surfaces and can easily and quickly detach from these surfaces at any time. Numerous studies [48,49,50] have investigated the attachment processes of insects; however, we believe that understanding the mechanical mechanisms underlying the detachment of insects from natural surfaces is equally important.

We believe that a subsequent study of the mechanics of the formation of intermolecular chemical bonds at the contact interface and their disruption by physical or chemical means during the process of insect adhesion and detachment from plant surfaces could be based on the Thomson model [51] used to study crack growth in solids. However, this model is outside of the scope of the present paper. This research will be the subject of subsequent papers. Detachment in engineering applications, such as robots [52,53,54], can occur when the surfaces or materials involved degrade or lose their substance. This can lead to failures at joints, connections, or adhesion points, affecting the performance of the robot. Due to the inherent adaptive capabilities of living organisms to respond to various adhesion–detachment conditions, many researchers [55,56,57] have embarked on algorithmic research to achieve bio-inspired robots with capacities for learning and adaptability comparable to those of biological entities. However, there is still a lack of research on the mechanical principles underlying the detachment process in living organisms. We hope that our subsequent research can provide theoretical support for applications such as bio-inspired climbing robots and grasping robotic arms in achieving the functionality of firm adhesion and rapid detachment.

The connection between structure and function is of great significance for the optimization of biomimetic surfaces. We have reviewed available information about the main mechanical properties of plant waxes and the possible fracture mechanisms of these wax structures during their interactions with insects. In particular, we have presented case studies concerning the mechanical analysis of columnar plant wax crystals with different aspect ratios under various load conditions. It is argued that plant surface microstructures, such as epicuticular waxes, significantly influence insect adhesion.

## Figures and Tables

**Table 1:** Mechanical properties of *carnauba* wax at two different temperatures [34].

Temperature	E (×10^6^ Pa)	$\sigma_{pr}\;(\times 10^{6}\mathrm{Pa})$	$\sigma_{st}\;(\times 10^{6}\mathrm{Pa})$
23 °C	1806.5	10.94	18.775
37 °C	772.2	5.72	9.03

**Table 2:** Geometrical parameters of the wax columns in selected plant species: *L*—length; *D*—diameter; *λ*—slenderness ratio.

Plant Species	*Aquilegia vulgaris*	*Berberis* *vulgaris*	*Chelidonium majus*	*Prunus* *domestica*	*Aristolochia fimbriata*
L(nm)	580	730	830	580	6310
D(nm)	170	160	180	260	92
$\lambda =L_{ef}/r$	8.18	10.94	11.06	5.35	164.6

**Table 3:** Yield moments of different plant wax crystals.

Plant Species	*Aquilegia vulgaris*	*Berberis* *vulgaris*	*Chelidonium majus*	*Prunus* *domestica*	*Aristolochia fimbriata*
D(nm)	170	160	180	260	92
$M_{pl}\left(\times 10^{-15}\;N\cdot m\right)$	28.7	23.9	34.0	102.5	4.5
$M_{p}\left(\times 10^{-15}\;N\cdot m\right)$	43	39	51	154	6.8

**Table 4:** Bending moments applied by insects to plant wax crystals.

Plant Species	*Aquilegia vulgaris*	*Berberis* *vulgaris*	*Chelidonium majus*	*Prunus* *domestica*	*Aristolochia fimbriata*
L(nm)	580	730	830	580	6310
$M_{L}\left(\times 10^{-15}\;N\cdot m\right)$	91	115	131	91	994

## Data Availability

The data used to support the findings of this study are available from the corresponding author upon request.
